# Heparanase Loosens E-Cadherin-Mediated Cell-Cell Contact via Activation of Src

**DOI:** 10.3389/fonc.2020.00002

**Published:** 2020-01-22

**Authors:** Victoria Cohen-Kaplan, Neta Ilan, Israel Vlodavsky

**Affiliations:** Rappaport Faculty of Medicine, Technion Integrated Cancer Center (TICC), Technion, Haifa, Israel

**Keywords:** heparanase, E-cadherin, Src, phosphorylation, cell migration

## Abstract

Activity of heparanase, responsible for cleavage of heparan sulfate (HS), is strongly implicated in tumor metastasis. This is due primarily to remodeling of the extracellular matrix (ECM) that becomes more prone to invasion by metastatic tumor cells. In addition, heparanase promotes the development of blood and lymph vessels that mobilize disseminated cells to distant organs. Here, we provide evidence for an additional mechanism by which heparanase affects cell motility, namely the destruction of E-cadherin based adherent junctions (AJ). We found that overexpression of heparanase or its exogenous addition results in reduced E-cadherin levels in the cell membrane. This was associated with a substantial increase in the phosphorylation levels of E-cadherin, β-catenin, and p120-catenin, the latter recognized as a substrate of Src. Indeed, we found that Src phosphorylation is increased in heparanase overexpressing cells, associating with a marked decrease in the interaction of E-cadherin with β-catenin, which is instrumental for AJ integrity and cell-cell adhesion. Notably, the association of E-cadherin with β-catenin in heparanase overexpressing cells was restored by Src inhibitor, along with reduced cell migration. These results imply that heparanase promotes tumor metastasis by virtue of its enzymatic activity responsible for remodeling of the ECM, and by signaling aspects that result in Src-mediated phosphorylation of E-cadherin/catenins and loosening of cell-cell contacts that are required for maintaining the integrity of epithelial sheets.

## Introduction

Heparan sulfate proteoglycans (HSPGs) consist of a protein core to which several linear heparan sulfate (HS) chains are covalently linked to specific serine residues. HSPGs bind to and assemble extracellular matrix (ECM) proteins (i.e., laminin, fibronectin, collagen type IV) and thereby contribute significantly to the physical (insolubility) and biological properties of the ECM (1–6). In addition, transmembrane (syndecans) and phospholipid-anchored (glypicans) HSPGs have a co-receptor role in which the proteoglycan, in concert with other cell surface molecules, comprises a functional receptor complex that facilitates signal transduction (1–3). The ECM provides an essential physical barrier between cells and tissues, plays an important role in cell growth, migration, differentiation and survival (7), and undergoes continuous remodeling during development and in certain pathological conditions such as wound healing and cancer (7, 8). ECM remodeling enzymes are thus expected to have a profound effect in many biological settings.

Heparanase is an endo-β-D-glucuronidase capable of cleaving HS side chains at a limited number of sites (9, 10). Heparanase activity is strongly implicated in tumor metastasis, a consequence of remodeling the ECM underlying epithelial cells (9–11). Similarly, heparanase activity was found to promote the motility of vascular endothelial cells and activated cells of the immune system (12–16). HS also bind a multitude of growth factors, chemokines, cytokines, and enzymes, thereby functioning as a low-affinity storage depot (17). Cleavage of HS side chains by heparanase is therefore expected not only to alter the integrity of the ECM but also to release HS-bound biological mediators that can function locally in a highly regulated manner. Intense research effort in the last two decades revealed that heparanase expression is often increased in human tumors (18, 19). In many cases, heparanase levels correlate with increased tumor metastasis, vascular density, and shorter postoperative survival of cancer patients (14, 16, 18, 20), thus providing strong clinical support for the pro-tumorigenic function of the enzyme and encouraging the development of heparanase inhibitors as anti-cancer drugs (21, 22). The pro-metastatic function of heparanase is attributed primarily to the cleavage of HS and remodeling of the ECM. In addition, heparanase promotes tumor vascularization (blood and lymph vessels) that mobilize disseminating cells to distant organs. Here, we show that heparanase disrupts adherent junctions (AJ) by augmenting the phosphorylation of E-cadherin and catenin family members (β-catenin, p120-catenin) that play an instrumental role in epithelial sheet adhesion, integrity, and function. This is mediated via increased Src phosphorylation in response to heparanase because treatment of heparanase overexpressing cells with Src inhibitors restored AJ, resulting in decreased cell migration. These results reveal another mechanism utilized by heparanase to promote cell dissemination and tumor metastasis.

## Materials and Methods

### Antibodies and Reagents

Anti E-cadherin (sc-8426), anti β-catenin (sc-7199), anti-paxillin (sc-5574), anti Src (sc-18 and sc-19), and anti-phosphotyrosine (sc-7020) antibodies were purchased from Santa Cruz Biotechnology (Santa Cruz, CA); Polyclonal antibody to phospho-Src (Tyr416) was purchased from Cell Signaling (Beverly, MA). Anti-actin and anti-ɤ-catenin (plakoglobin) antibodies were purchased from Sigma (St. Louis, MO). Anti p120-catenin was purchased from Becton Dickinson (Mountain View, CA); Anti heparanase polyclonal antibody (#1453) has been described previously (23). The selective Src (PP2) and EGFR (CL-387,785) inhibitors were purchased from Calbiochem (San Diego, CA) and were dissolved in DMSO as stock solutions. DMSO was added to the cell culture as control. Phalloidin-TRITC and streptavidin-HRP were purchased from Sigma.

### Cell Culture and Transfection

FaDu pharynx carcinoma cells were kindly provided by Dr. Eben L. Rosenthal (the University of Alabama at Birmingham, Birmingham, AL) (24); JSQ3 nasal vestibule carcinoma cells were kindly provided by Dr. Ralph Weichselbaum (University of Chicago, Chicago, IL) (25); SIHN-013 laryngeal carcinoma cells were kindly provided by Dr. Sue Eccles (Institute of Cancer Research, Sutton, Surrey, UK) (26); T47D breast carcinoma cells were purchased from the American Type Culture Collection (ATCC). Cells were cultured in Dulbecco's Modified Eagle's (DMEM) or RPMI medium (T47D) supplemented with glutamine, pyruvate, antibiotics and 10% fetal calf serum in a humidified atmosphere containing 5% CO_2_ at 37°C. For stable transfection, cells were transfected with heparanase gene constructs using the FuGene reagent according to the manufacturer's (Roche) instructions, selected with Zeocin (Invitrogen, Carlsbad, CA) for 2 weeks, expanded and pooled, as described (27, 28). Cells were passed in culture for no more than 3 months after being thawed from authentic stocks.

HEK 293 cells, stably transfected with the human heparanase gene construct in the mammalian pSecTag vector (Invitrogen), were kindly provided by ImClone Systems (New York, NY). The cells were grown in DMEM supplemented with 10% FCS, glutamine, pyruvate, and antibiotics. For heparanase purification, the cells were grown overnight in serum-free-DMEM and the conditioned medium (~1 liter) was purified on a Fractogel EMD SO3^−^ (MERCK) column. The bound material was eluted with 1 M NaCl and was further purified by affinity chromatography on anti-c-Myc (Santa Cruz Biotechnology) column. We obtained at least 95% pure heparanase preparation by this two-step procedure (29).

### Cell Fractionation, Immunoprecipitation, and Protein Blottin

Isolation of plasma membrane fraction was carried out essentially as described (30). Briefly, T47D cells (3 × 10^8^) were harvested by EDTA (2.5 mM), washed twice with PBS, suspended in 1 ml extraction buffer (10 mM Tris/acetic acid buffer, pH 7.0, supplemented with 250 mM sucrose) and were incubated for 20 min on ice. Cells were then homogenized in 5 ml Potter-Elvehjen homogenizer followed by centrifugugation at 2,000 × g for 2 min; The supernatant was collected and centrifuged at 4,000 x g for 2 min to pellet a fraction enriched with plasma membranes. Membrane proteins were dissolved with lysis buffer (50 mM Tris-HCl, pH 7.4, 150 mM NaCl, 1% Triton-X100, 1 mM orthovanadate, 1 mM PMSF) and equal amounts of protein were subjected to immunoblotting.

Preparation of cell lysates, immunoprecipitation, and immunoblotting was performed essentially as described (27, 28). Briefly, cell cultures were pretreated with 1 mM orthovanadate for 10 min at 37°C, washed twice with ice-cold PBS containing 1 mM orthovanadate and scraped into lysis buffer (50 mM Tris-HCl, pH 7.4, 150 mM NaCl, 0.5% NP-40, 1 mM orthovanadate, 1 mM PMSF) containing a cocktail of proteinase inhibitors (Roche). Total cellular protein concentration was determined by the BCA assay according to the manufacturer's instructions (Pierce, Rockford, IL). Thirty μg of cellular protein were resolved on SDS polyacrylamide gel, and immunoblotting was performed, as described (23, 29). Immunoblots were subjected to densitometry analyses and the relative intensity of bands (i.e., fold change) is presented underneath the gel. Changes in protein phosphorylation is presented in comparison to control (Vo) cells, set arbitrarily to a value of 1, and following normalization to the total levels of the protein in the cell lysate. Immunoprecipitation (IP) was carried out essentially as described (31). Briefly, 600 μg of cellular protein were brought to a volume of 1 ml in buffer containing 50 mM Tris-HCl, pH 7.5, 150 mM NaCl, and 0.5% NP-40, incubated with the appropriate antibody for 4 h on ice followed by incubation with protein G-Sepharose (Rosche; 60 min on ice). Beads were washed twice with the same buffer supplemented with 5% sucrose. Sample buffer was added, and samples were boiled and subjected to gel electrophoresis and immunoblotting, as described above.

### Surface Biotinylation

Surface biotinylation was carried out by using EZ link Sulfo-NHS-SS-Biotin according to the manufacture's (Thermo Fisher Scientific) instructions. Briefly, Sulfo-NHS-SS-Biotin was dissolved in PBS containing Ca++ and Mg++ to a concentration of 0.5 mg/ml and added to cell culture for 30 min on ice. Cell culture was then washed (×3) with ice-cold quenching solution (50 mM glycine in PBS containing Ca++ and Mg++). Cell lysates were then prepared and subjected to IP for E-cadherin, followed by immunoblotting with streptavidin-HRP (Sigma).

### Immunocytochemistry

Immunofluorescent staining was performed essentially as described (23, 27, 32). Briefly, cells were grown on glass coverslips for 18 h. Heparanase (1 μg/ml) was then added for the time indicated, cells were washed with PBS and fixed with 4% paraformaldehyde (PFA) for 20 min. Cells were then permeabilized for 1 min with 0.5% Triton X-100, washed with PBS and incubated in PBS containing 10% normal goat serum for 1 h at room temperature, followed by 2 h incubation with the indicated primary antibody. Cells were then extensively washed with PBS and incubated with the relevant Cy2/Cy3-conjugated secondary antibody (Jackson ImmunoResearch, West Grove, PA) for 1 h, washed and mounted (Vectashield, Vector, Burlingame, CA). Wound healing migration assay was carried out essentially as described (29).

### Flow Cytometry

Cells were detached with 2.5 mM EDTA, centrifuged at 1000 RPM for 4 min., washed with PBS and counted. Cells (2 × 10^5^) were centrifuged and the pellet was then resuspended in PBS containing 1% FCS and incubated with FITC conjugated anti-E-cadherin antibody for 40 min on ice. Cells were then washed twice with PBS and analyzed using a FACSCalibur fluorescent activated cell sorter and CellQuest software (Becton Dickinson, Mountain View, CA), as described (29).

### Statistics

Results are shown as means ±SE. GraphPad Instat software was used for statistical analysis. The differences between the control and treatment groups were determined by two-tailed Student's *t*-Test. Statistically significance is presented according to the common use of ^*^p < 0.05; ^**^p < 0.01; ^***^p < 0.001.

## Results

### Heparanase Disrupts Adherent Junctions (AJ)

**Figure 1:**
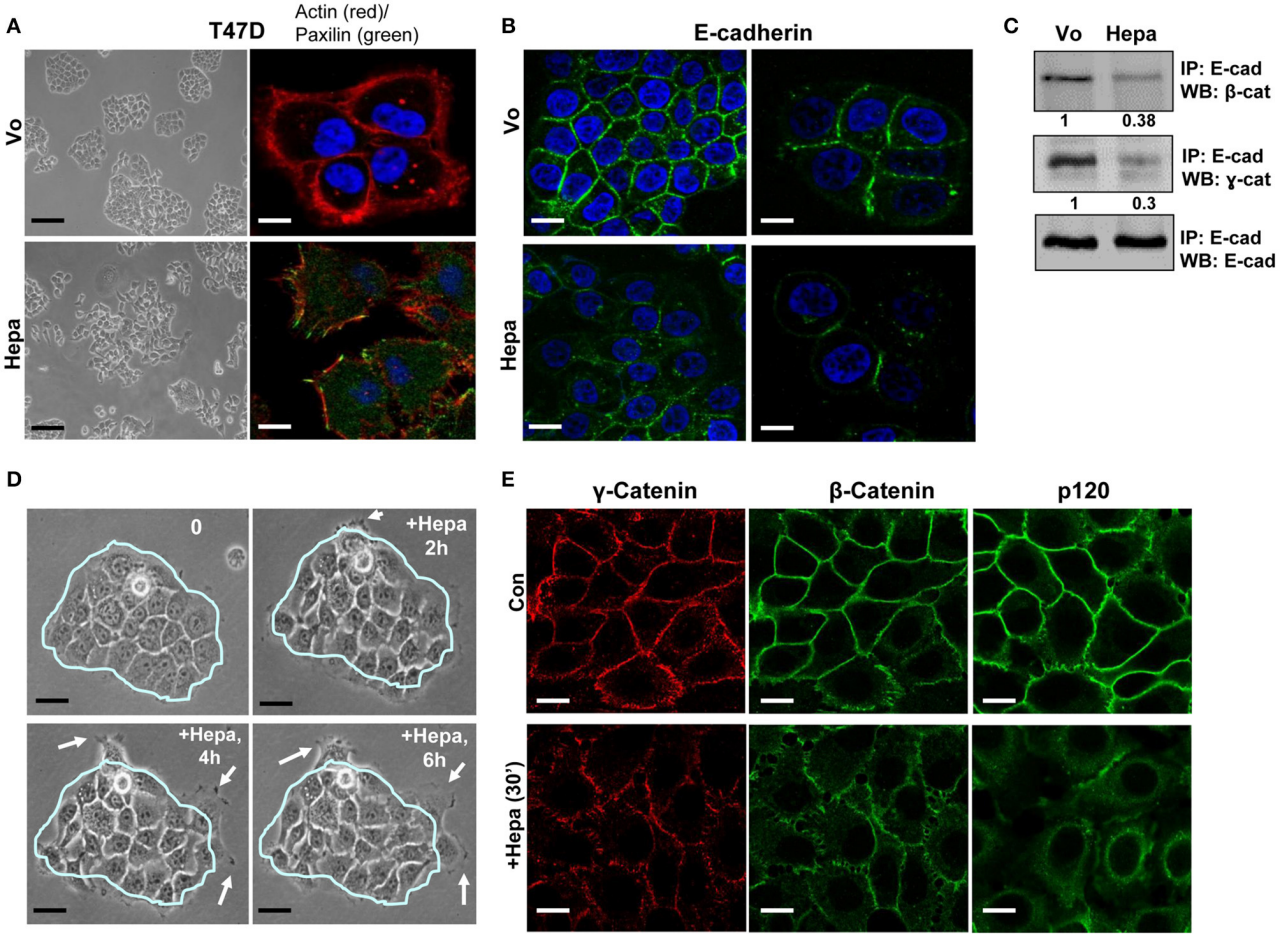
Heparanase affects cell-cell contacts and disrupts AJ. **(A)** Dispersed cell colonies. T47D breast carcinoma cells were transfected with an empty vector (Vo) or heparanase gene construct (Hepa), and their growth pattern was examined. Shown are representative cell cultures. Note that while control (Vo) cells grew in typical well-organized colonies, heparanase overexpressing cells show dispersed cell colonies (left panels). Control (Vo) and heparanase overexpressing cells were fixed with 4% PFA and following permeabilization were stained with phalloidin-TRITC to label the actin cytoskeleton (red), and paxillin (green) that typically labels adherent junctions (right panels). Note abundant paxillin staining in heparanase overexpressing cells. Scale bars represent 60 (left panels) and 10 microns (right panels). **(B)** Decreased E-cadherin staining in heparanase cells. Control (Vo) and heparanase overexpressing cells (Hepa) were subjected to immunofluorescent staining applying anti-E-cadherin antibody. Shown are representative images (confocal microscopy) at ×100 (left) and ×200 (right) magnifications merged with nuclear labeling (DAPI; blue). Note decreased E-cadherin at cell-cell borders upon heparanase overexpression. Scale bars represent 20 (left panels) and 10 (right panels) microns. **(C)** Immunoprecipitation. Lysates of control (Vo) and heparanase overexpressing cells (Hepa) were subjected to IP applying anti-E-cadherin antibody, followed by immunoblotting with anti-β-catenin (upper panel), ɤ-catenin (second panel), and anti-E-cadherin (lower panel) antibodies. Densitometry analysis of protein band intensity is shown below each panel in relation to its level in control (Vo) cells, set arbitrarily to a value of 1. Note decreased association of E-cadherin with catenins in heparanase cells. **(D)** Exogenous addition. T47D cells were seeded at low density, and cell colonies were allowed to form. Colonies were then photographed and their morphology was inspected over time following treatment with latent heparanase added exogenously (1 μg/ml) to the cell culture medium. Shown is a typical colony before (0) and after the addition of heparanase for 2, 4, and 6 h. Note that cells are migrating out of the colony (white arrows) after the addition of heparanase. Scale bars represent 30 microns. **(E)** Immunofluorescent staining. T47D cells were left untreated (Con) or were treated for 30 min with latent heparanase (1 μg/ml) added exogenously to the cell culture medium. Cells were then fixed, permeabilized, and subjected to immunofluorescent staining applying anti-ɤ-catenin (left panels), anti-β-catenin (middle panels), and anti-p120-catenin (right panels) antibodies. Note decreased and less organized staining of the catenins following the addition of heparanase. Scale bars represent 10 microns.

Heparanase expression is often induced in carcinomas and is associated with increased tumor metastasis and bad prognosis (19, 33), but the effect of heparanase on AJ has not been reported yet. We noticed that overexpression of heparanase in T47D breast carcinoma cells resulted in more dispersed cell colonies (Figure 1A, left). These cells also exhibited more abundant focal contacts evident by paxillin staining (Figure 1A, right), typical of migrating cells. A similar increase in paxillin staining was observed following exogenous addition of latent heparanase (65 kDa) to SIHN-013 laryngeal and JSQ3 nasal vestibule carcinoma cells (Supplementary Figure 1A). Notably, overexpression of heparanase was associated with decreased E-cadherin at cell-cell borders evident by immunofluorescent staining (Figure 1B), cell surface biotinylation (Supplementary Figure 1B, upper panel), and immunoblotting of cell membrane fractions (Supplementary Figure 1B, lower panel). Moreover, overexpression of heparanase was associated with a decreased interaction (3-fold) of E-cadherin with β- and ɤ-catenin (Figure 1C) which is essential to connect E-cadherin with the actin cytoskeleton and establish functional AJ. Increased migration of cells out of well-organized colonies was observed following exogenous addition of latent heparanase protein (Figure 1D) and is best demonstrated by time-lapse microscopy (Supplementary Videos 1, 2). Reduced levels of β-, ɤ-, and p120-catenin at cell-cell borders were evident already 30 min after the addition of heparanase, and the catenins that were retained on the cell surface appeared discontinued and were arranged in a patchy manner (Figure 1E, Supplementary Figure 1C, left and middle panels). The rapid decrease of E-cadherin/catenins from cell-cell borders may suggest the involvement of a signaling pathway elicited by heparanase.

### Disruption of AJ by Heparanase Is Mediated by Src

**Figure 2:**
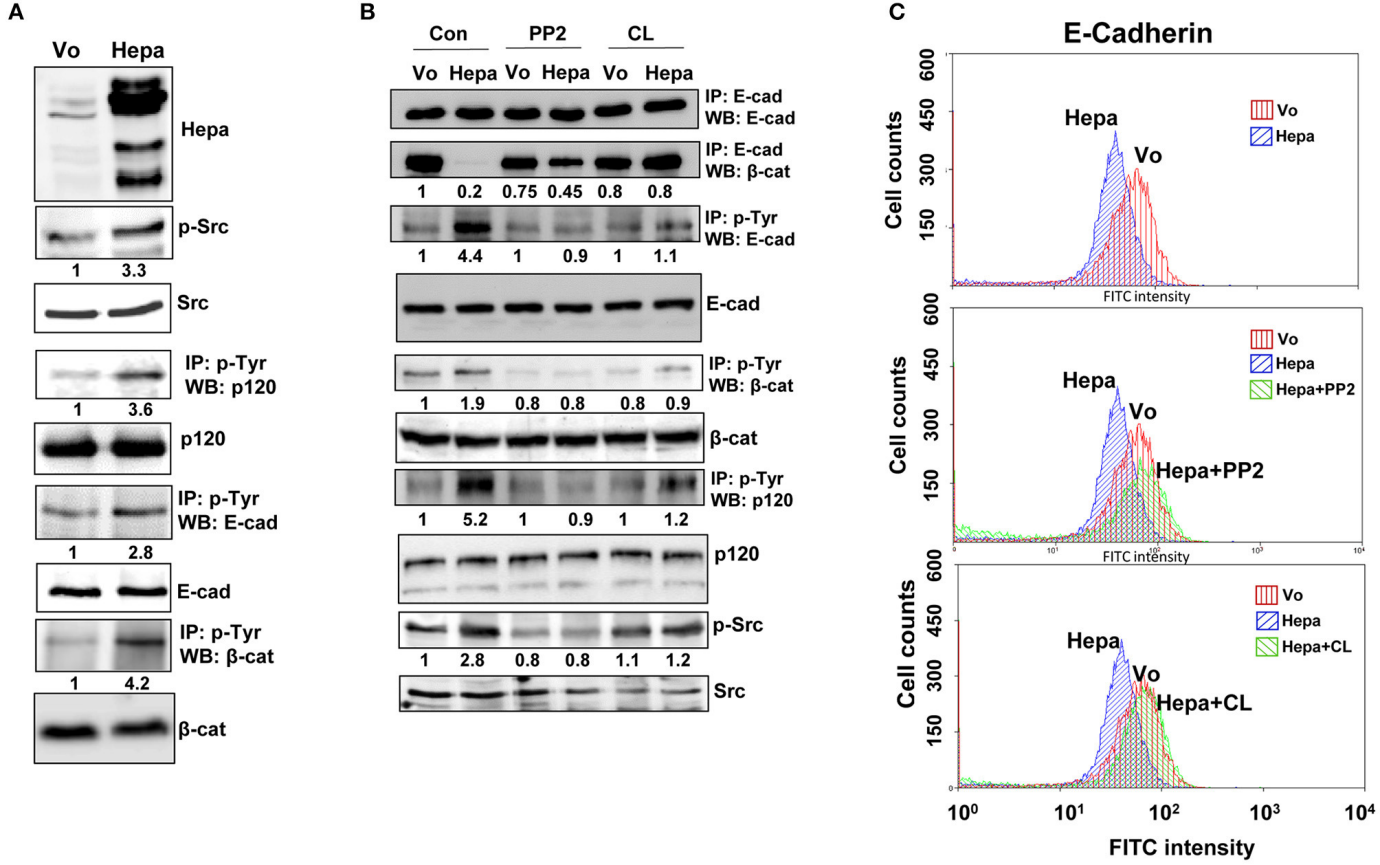
Heparanase enhances the phosphorylation of E-cadherin and catenins via activation of Src. **(A)** immunoblotting. Lysates of control (Vo) and heparanase (Hepa) overexpressing cells were subjected to immunoblotting applying anti-heparanase (upper panel), anti-phospho–Src (p-Src; second panel), and anti-Src (third panel) antibodies. Cell lysates were subjected to IP with anti-phospho-tyrosine antibody (p-Tyr), followed by immunoblotting with anti-p120-catenin (fourth panel), anti-E-cadherin (sixth panel), and anti-β-catenin (eighth panel) antibodies. Densitometry analysis of protein band intensity is shown below each panel in relation to its level in control (Vo) cells, set arbitrarily to a value of 1, and following normalization to the total levels of Src, p120, E-Cadherin and β-catenin (third, fifth, seventh, and ninth panels, respectively) in the cell lysates. **(B)** Inhibitors of Src and EGFR restore the association of E-cadherin with β-catenin. Control (Vo) and heparanase overexpressing cells (Hepa) were treated with vehicle (DMSO) as control (Con) or with inhibitors of Src (PP2; 5 μM) or EGFR (CL-387,785; 0.01 μM) for 3 h. Cell lysates were then prepared and subjected to IP with anti-E-cadherin antibody, followed by immunoblotting with anti-E-cadherin (upper panel) and anti-β-catenin (second panel) antibodies. Lysates were similarly subjected to IP with anti-phosphotyrosine (p-Tyr) antibody, followed by immunoblotting with anti-E-cadherin (third panel), anti-β-catenin (fifth panel), and anti-p-120-catenin (seventh panel) antibodies. Cell lysates were similarly immunoblotted applying anti-phospho-Src (p-Src; ninth panel) and anti-Src (lower panel) antibodies. Densitometry analysis of protein band intensity is shown below each panel in relation to its level in control (Vo) cells, set arbitrarily to a value of 1, and following normalization to the total levels of E-Cadherin, β-catenin, p120, and Src (fourth, sixth, eighth, and tenth panels, respectively) in the cell lysates. Corresponding control (Vo) and Hepa cells treated with DMSO (vehicle), PP2, or CL-387,785 were detached with EDTA and subjected to FACS analyses applying anti-E-cadherin antibody **(C)**. Note that inhibition of Src or EGFR restores the association of E-cadherin with β-catenin in Hepa cells.

We have reported previously that overexpression of heparanase augments the phosphorylation levels of p120-catenin (34), a catenin-family member originally identified as a Src substrate (35). Indeed, overexpression of heparanase in T47D cells (Figure 2A, upper panel) was associated with increased phosphorylation levels of Src (3.3-fold; Figure 2A, second panel) and p120-catenin (3.6-fold; Figure 2A, fourth panel), in agreement with earlier reports showing that heparanase enhances Src phosphorylation (27, 34, 36). Similarly, the phosphorylation levels of E-cadherin and β-catenin were also augmented substantially in cells overexpressing heparanase (2.8- and 4.2-fold, respectively; Figure 2A, sixth and eighth panels). Given that E-cadherin/catenins phosphorylation results in the dissociation of AJ (37, 38), we investigated whether Src inhibitors (e.g., PP2) would restore AJ integrity in cells overexpressing heparanase. To this end, control (Vo) and heparanase (Hepa) cells were treated with DMSO as vehicle control (Con) or with PP2, and cell extracts were subjected to IP for E-cadherin. While the total levels of E-cadherin appeared similar in control (Vo) and heparanase (Hepa; Figure 2B, upper panel) cells, its association with β-catenin was strikingly lower in heparanase overexpressing cells (Hepa; Figure 2B, second panel, Con), but was increased prominently in heparanase cells treated with PP2 (PP2; Figure 2B, second panel). Likewise, PP2 treatment was associated with a marked decrease in the phosphorylation levels of E-cadherin (PP2; Figure 2B, third panel), β-catenin (PP2; Figure 2B, fifth panel), p120-catenin (PP2; Figure 2B, seventh panel), and Src (Figure 2B, ninth panel). Interestingly, similar results were obtained in cells treated with an inhibitor of the EGF receptor (EGFR), CL-387,785 (Figure 2B, CL). This may suggest that Src phosphorylates and activates the EGFR (27), leading to the disruption of AJ (39).

**Figure 3:**
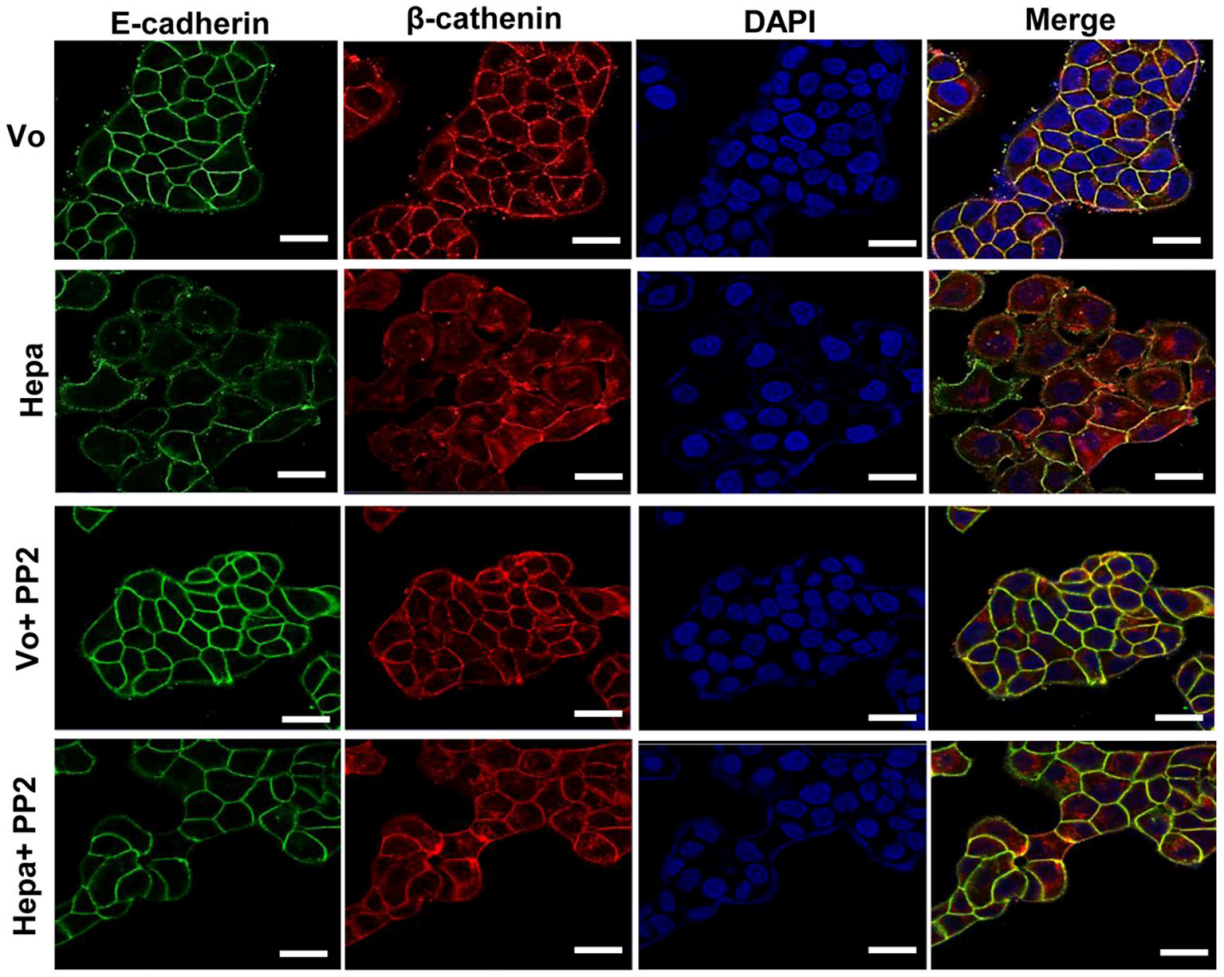
Immunofluorescent staining. Control (Vo) and heparanase overexpressing T47D cells (Hepa) were left untreated or were treated with PP2 (5 μM) for 3 h. Cells were then fixed with 4% PFA, permeabilized, and subjected to immunofluorescent staining applying anti-E-cadherin (green) and anti-β-catenin (red) antibodies. Merged images are shown in the right panels together with nuclear counterstaining (blue). Shown are representative images (confocal microscopy) at ×100 magnification. Note that far more E-cadherin and β-catenin are recruited to cell-cell contacts following Src inhibition with PP2. Scale bars represent 15 microns.

In order to further reveal the restoration of AJ by Src inhibitor evident by co-IP (Figure 2B), we subjected control and PP2 treated cells to FACS analyses. While the levels of E-cadherin at the cell surface was decreased in cells overexpressing heparanase vs. control (Vo) cells (Figure 2C, upper panel), in agreement with the surface biotinylation and membrane fractionation approaches (Supplementary Figure 1B), treatment with PP2 (Figure 2C, second panel) and CL-387,785 (Figure 2C, lower panel) restored its localization at the cell surface to the levels of control (Vo) cells. This was further evident by immunofluorescent staining (Figure 3), clearly depicting that treatment of heparanase overexpressing cells with PP2 results in recruitment of E-cadherin to the cell surface and restoration of AJ.

### Heparanase Promotes Cell Migration via Activation of Src

**Figure 4:**
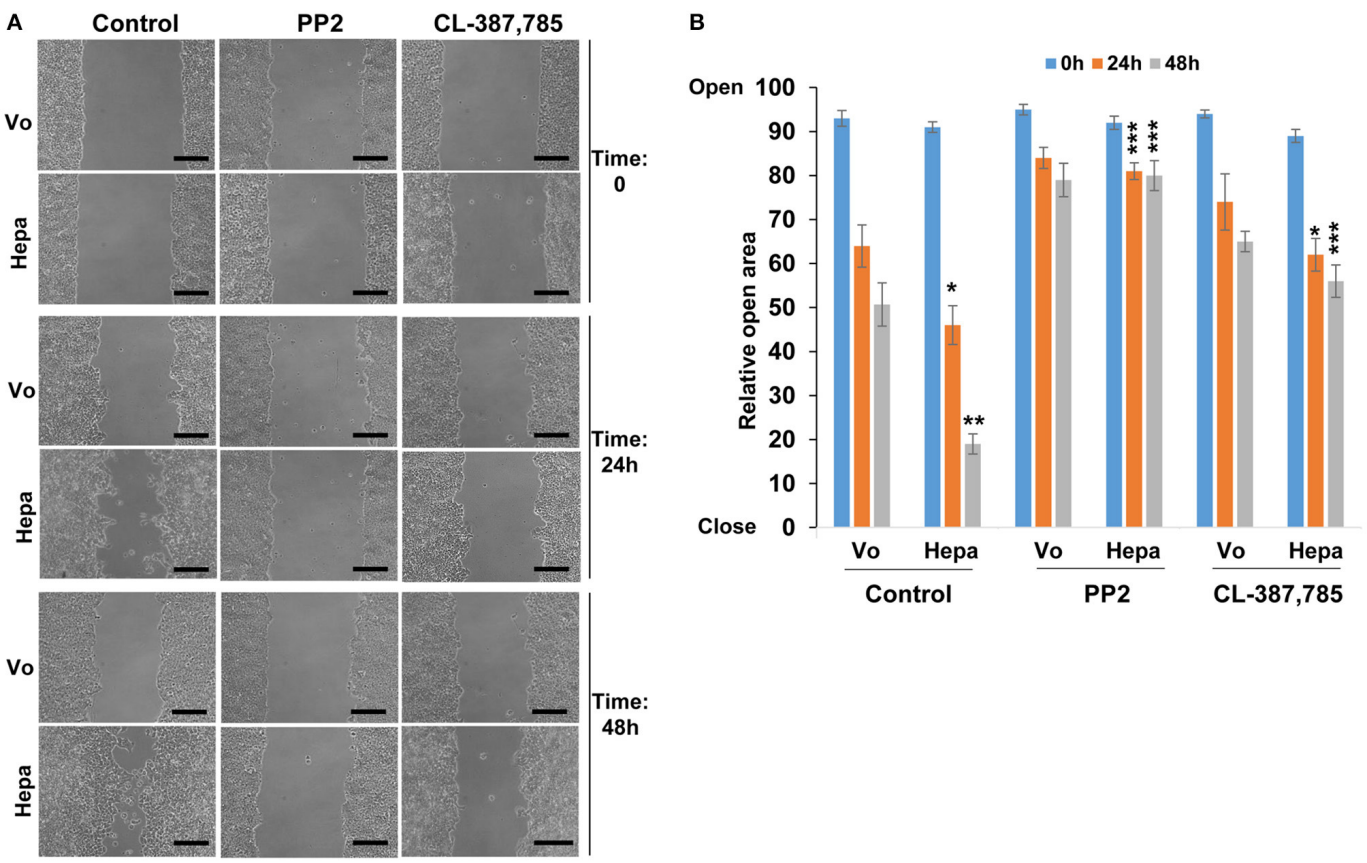
Cell migration. Control (Vo) and heparanase overexpressing (Hepa) T47D cells were grown to confluence. Cultures were then scratched and were treated with DMSO (vehicle) as control or with PP2 (5 μM) or CL-387,785 (0.01 μM). Cell migration into the wounded area was observed over 2 days. Shown are representative images taken immediately after wounding (Time 0), and 24 and 48 h thereafter **(A)**. Quantification of relative wound closure is shown graphically in **(B)**. Note that heparanase overexpressing cells fill the wounded area faster than control cells and this increase in cell migration is reversed by Src- and to a lesser extent by EGFR- inhibitor. Scale bars represent 150 microns.

Cell-cell contact and AJ integrity play an instrumental role in cell migration. To examine the consequences of increased E-cadherin/catenins phosphorylation in heparanase overexpressing cells and the associated disruption of AJ on cell migration, we employed a wound-healing assay. We found that heparanase cells migrate faster than control (Vo) cells. This was evident already 24 h post wounding (Control; Figures 4A,B, 24 h; *p* < 0.05 for Vo vs. Hepa), and became most evident by 48 h when heparanase cells filled the wounded area almost completely (Control; Figure 4A, lower panels & Figure 4B; *p* < 0.01 for Vo vs. Hepa). Importantly, the pro-migratory function of heparanase was abrogated by inhibitors of Src (PP2; Figures 4A,B; *p* < 0.001 for Hepa vs. Hepa+PP2 at 24 and 48 h) and EGFR (CL; Figures 4A,B; *p* < 0.05 and *p* < 0.001 for Hepa vs. Hepa+CL at 24 and 48 h, respectively), along with the restoration of AJ (Figures 2B,C, 3), further signifying that heparanase promotes cell migration by activation of Src, leading to disruption of E-cadherin-based cell-cell contact.

## Discussion

Heparanase has long been implicated in tumor metastasis. This notion is now well-accepted and supported by compelling pre-clinical and clinical data (19, 20, 33, 40). The pro-metastatic function of heparanase is largely attributed to its enzymatic activity capable of cleaving HS and, consequently, remodeling of the ECM underlying epithelial and endothelial cells. In addition, heparanase enhances the formation of new blood and lymph vessels (19, 20, 28, 33, 40), thereby promoting the mobilization of disseminating tumor cells to distant organs. Here, we describe a new mechanism by which heparanase can promote cell dissemination namely, disruption of AJ. E-cadherin-based AJ are characteristic of all epithelial cells. Through the homophilic association of E-cadherin molecules expressed on neighboring cells, they ensure intercellular adhesion between epithelial cells and regulate many key aspects of epithelial biology (37). AJ structures are stabilized by the accumulation of a dense actin filaments-based network, mediated by anchoring E-cadherin clusters to the inner cytoskeleton. The link to the actin cytoskeleton is mainly mediated by β-catenin via its association with α-catenin (37, 38). In mammalian cells, the E-cadherin/catenin complex and AJ stability are tightly regulated by phosphorylation, where Src kinase and Src-family members are thought to play an instrumental role (37–39). More specifically, phosphorylation of β-catenin by Src results in reduced association with E-cadherin and α-catenin, leading to AJ disruption and subsequent decreased cell-cell adhesion (37–39). Importantly, such a decrease in cell-cell contacts and loss of E-cadherin has been associated with advanced tumor stages and poor prognosis in patients with cancer (38, 41).

Previously, we have reported that heparanase enhances the phosphorylation of Src, associating with increased cell proliferation and colony formation in soft agar (27, 34, 36). The mechanism by which heparanase enhances the phosphorylation of Src is not entirely clear, but seems to be independent of heparanase enzymatic activity. This was concluded because increased Src phosphorylation was observed in cells over expressing heparanase that was mutated in glutamic acids 225 and 343 that comprise the enzyme active site (42), or heparanase that was deleted for the heparin binding domain [amino acids 270–280; Δ10; (31)] (27, 36), indicating that Src activation does not require heparanase enzymatic activity or its interaction with HS. Thus, inhibitors of heparanase activity such as HS-mimetics or JG6, a marine-derived oligosaccharide (43, 44), are not expected to attenuate this function of heparanase. It is possible, nonetheless, that Src activation is downstream to the activation of the epidermal growth factor receptor (EGFR) (27), focal adhesion kinase (FAK) (44), or integrin (29) by heparanase. Activation of Src family members such as Fyn, Lyn, or Hck by heparanase has not been so far reported. Here, we confirm and further expand the consequences of Src activation by heparanase. Notably, overexpression of heparanase in T47D cells was associated with increased Src phosphorylation, more dispersed cell colonies (Figure 1A, Supplementary Videos 1, 2), and decreased E-cadherin at cell-cell borders. This was evident by immunofluorescent staining (Figure 1B), FACS analyses (Figure 2C), surface biotinylation (Supplementary Figure 1B, upper panel), and immunoblotting of membrane fractions (Supplementary Figure 1B, lower panel). Moreover, the phosphorylation levels of E-cadherin, p120-catenin, and β-catenin were increased markedly in cells overexpressing heparanase (Figures 2A,B), modifications that are highly associated with disruption of AJ (38, 41). Indeed, IP experiments revealed a remarkable decrease in the association of E-cadherin with β-catenin (Figure 2B), which was restored in heparanase cells treated with Src inhibitor (PP2; Figure 2B). Similarly, localization of E-cadherin to the cell membrane, evident by FACS analyses and immunofluorescent staining, was increased in heparanase cells treated with PP2 (Figures 2C, 3). Disruption of AJ typically leads to reduced cell-cell contacts and increased cell migration. Indeed, heparanase was noted to promote cell adhesion and cell migration in a manner that seems not to involve its enzymatic activity (29, 32, 45, 46). Our results suggest that increased cell migration by heparanase involves Src-mediated phosphorylation of E-cadherin/catenins. This notion is supported by the observed increased cell migration and wound closure of T47D cells overexpressing heparanase, and decreased wound closure following treatment with PP2 (Figure 4). Importantly, treatment of mice with PP2 for 3 weeks markedly reduced the rate of liver metastasis by colon carcinoma cells (47), thus signifying the critical role of Src in disrupting AJ integrity, leading to cell dissemination and tumor metastasis. Reduced E-cadherin expression is often observed in the context of epithelial-mesenchymal transition (EMT), accompanied by increased levels of mesenchymal proteins such as N-cadherin, vimentin, and fibronectin (48). We did not observe changes in the expression levels of E-cadherin upon heparanase overexpression nor activation of an EMT program (i.e., induction of Twist, Snail, Slug, or ZEB transcription factors) (data not shown), suggesting that Src activation is the main force that drives E-cadherin/catenin phosphorylation and disruption of AJ. Notably, heparanase was found to elicit EMT in the context of kidney injury (49–52), suggesting that activation of the EMT program by heparanase can occur, depending on the biological context and experimental system employed.

## Data Availability Statement

The datasets generated for this study are available on request to the corresponding author.

## Author Contributions

IV and NI designed the research. VC-K performed the research. VC-K, IV, and NI analyzed the data and wrote the paper.

### Conflict of Interest

The authors declare that the research was conducted in the absence of any commercial or financial relationships that could be construed as a potential conflict of interest.

## Supplementary Material

Supplementary Figure 1 **(A)** Paxillin staining. Latent heparanase (1 μg/ml) was added exogenously to SIHN-013 laryngeal carcinoma (013; left) and JSQ3 nasal vestibule carcinoma (right) cells. After 2 h, cells were fixed with 4% PFA, permeabilized, and subjected to immunofluorescent staining applying anti-paxillin antibody (green) along with phalloidin-TRITC (red) staining. Note increased paxillin staining at focal contacts following the addition of heparanase. Scale bars represent 10 microns. **(B)** Localization of E-cadherin on the cell membrane is decreased in heparanase overexpressing cells. T47D cells were subjected to surface biotinylation as described under “Materials and Methods.” Cell extracts were then prepared and subjected to IP with anti-E-cadherin antibody, followed by immunoblotting with streptavidin-HRP (SA-HRP; upper panel) and anti-E-cadherin antibody (second panel). Control (Vo) and heparanase cells were subjected to cell fractionation as described in “Materials and Methods” and membrane fractions were subjected to immunoblotting applying anti-E-cadherin antibody (lower panel). Note reduced E-cadherin on the cell membrane of heparanase overexpressing cells. **(C)** Heparanase was added exogenously to FaDu cells for 4 h and the cells were then subjected to immunofluorescent staining applying anti-ɤ-catenin (left) and anti-β-catenin (middle) antibodies. JSQ3 nasal vestibule carcinoma cells were transfected with an empty vector (Vo) or heparanase gene construct (Hepa) and were subjected to immunofluorescent staining applying anti-β-catenin antibody. Scale bars represent 10 (left panels) and 30 (right panels) microns.

Supplementary Video 1 T47D breast carcinoma cells (2 × 10^4^) were plated in a 6-well plate in complete growth medium for 24 h. Cells were then serum starved for 6 h, six fields in each well were randomly selected and examined every 10 min for 18 h by a time-lapse system. Representative time-lapse movie is shown.

Supplementary Video 2 T47D breast carcinoma cells (2 × 10^4^) were plated in a 6-well plate in complete growth medium for 24 h. Cells were then serum starved for 6 h. Latent heparanase (1 μg/ml) was then added, six fields in each well were randomly selected and examined every 10 min for 18 h by a time-lapse system. Representative time-lapse movie is shown.
