# Supplementary figures and images for: Heparanase Loosens E-Cadherin-Mediated Cell-Cell Contact via Activation of Src
Source: Front Oncol. 2020 Jan 22;10:2. doi: 10.3389/fonc.2020.00002 (PMC6990126; doi:10.3389/fonc.2020.00002)

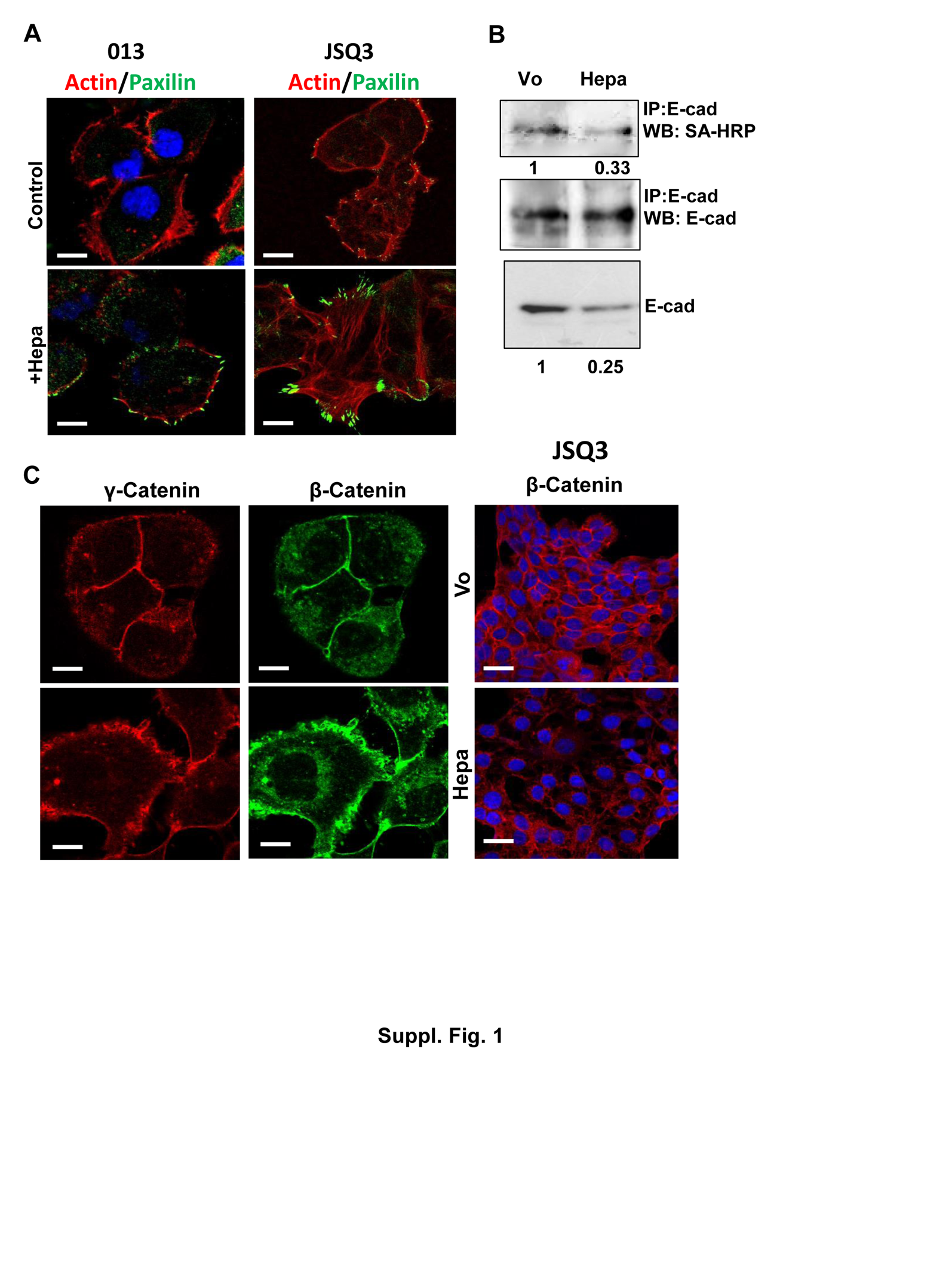

Supplement: Supplementary Figure 1 — (A) Paxillin staining. Latent heparanase (1 μg/ml) was added exogenously to SIHN-013 laryngeal carcinoma (013; left) and JSQ3 nasal vestibule carcinoma (right) cells. After 2 h, cells were fixed with 4% PFA, permeabilized, and subjected to immunofluorescent staining applying anti-paxillin antibody (green) along with phalloidin-TRITC (red) staining. Note increased paxillin staining at focal contacts following the addition of heparanase. Scale bars represent 10 microns. (B) Localization of E-cadherin on the cell membrane is decreased in heparanase overexpressing cells. T47D cells were subjected to surface biotinylation as described under “Materials and Methods.” Cell extracts were then prepared and subjected to IP with anti-E-cadherin antibody, followed by immunoblotting with streptavidin-HRP (SA-HRP; upper panel) and anti-E-cadherin antibody (second panel). Control (Vo) and heparanase cells were subjected to cell fractionation as described in “Materials and Methods” and membrane fractions were subjected to immunoblotting applying anti-E-cadherin antibody (lower panel). Note reduced E-cadherin on the cell membrane of heparanase overexpressing cells. (C) Heparanase was added exogenously to FaDu cells for 4 h and the cells were then subjected to immunofluorescent staining applying anti-ɤ-catenin (left) and anti-β-catenin (middle) antibodies. JSQ3 nasal vestibule carcinoma cells were transfected with an empty vector (Vo) or heparanase gene construct (Hepa) and were subjected to immunofluorescent staining applying anti-β-catenin antibody. Scale bars represent 10 (left panels) and 30 (right panels) microns. [file Image_1.TIF]
